# Thoracic Skeletal Muscle and Exercise Capacity in Adults With Congenital Heart Disease: A Cross‐Sectional Imaging Analysis

**DOI:** 10.1002/jcsm.70157

**Published:** 2025-12-08

**Authors:** Jacob D. Steffen, Seth Garton, Ravi Ashwath, Jennifer R. Maldonado, Krista Young, Colleen Lancial, Osamah Aldoss, Prashob Porayette

**Affiliations:** ^1^ Division of Pediatric Cardiology, Department of Pediatrics, Stead Family Children's Hospital University of Iowa Carver College of Medicine Iowa City Iowa USA; ^2^ Medical College of Wisconsin, Children's Wisconsin Milwaukee Wisconsin USA

**Keywords:** ACHD, congenital heart disease, exercise, sarcopenia, skeletal muscle

## Abstract

**Background:**

The growing population of adults with congenital heart disease (ACHD) faces lifelong morbidities despite advancements in medical and surgical care. Sarcopenia, characterized by loss of muscle mass and strength, is linked to increased disability, poor quality of life and mortality. This study examines sex‐specific thoracic skeletal muscle characteristics in ACHD patients using advanced imaging techniques, comparing them with healthy reference values and investigating their association with exercise capacity.

**Material and Methods:**

In this single‐centre retrospective study, ACHD patients (age 18–50 years) who underwent both cardiopulmonary exercise tests and thoracic CT/MRI within a year were included. Skeletal muscle area (SMA) was manually measured and compared with healthy reference data.

**Results:**

Among 60 ACHD patients (mean age 28.3 ± 8.3 years; 48% females), males exhibited significantly lower SMA (T10: 116.8 ± 24.6 cm^2^, *p* < 0.0001; T11: 114.4 ± 24.7 cm^2^, *p* = 0.0002) and skeletal muscle index (SMI) (T10: 37.2 ± 8 cm^2^/m^2^, *p* = 0.005; T11: 36.4 ± 8.1 cm^2^/m^2^, *p* = 0.0014) at T10 and T11 vertebral level, whereas females showed a reduction in SMA at T10 (79.7 ± 14.9 cm^2^, *p* = 0.0242) and T12 (74.2 ± 10.7 cm^2^, *p* = 0.0015) compared with healthy individuals. Females had significantly lower skeletal muscle radiation attenuation (SMRA) at T10 (16.3 ± 14.6 HU, *p* < 0.001), T11 (17.1 ± 10.3 HU, *p* < 0.001) and T12 (25 ± 10.7 HU, *p* < 0.001) levels, suggesting increased muscle fat content. Peak O_2_ pulse correlated with SMA at T10 (*r* = 0.57, *R* ^2^ = 0.32, *p* ≤ 0.0001), T11 (*r* = 0.61, *R* ^2^ = 0.38, *p* < 0.0001) and T12 (*r* = 0.73, *R* ^2^ = 0.53, *p* = 0.001) levels. Similar correlations were observed between peak O_2_ pulse and SMI, whereas peak VO_2_ correlated with SMA at T10 (*r* = 0.27, *R* ^2^ = 0.07, *p* = 0.0394) and T11 (*r* = 0.34, *R* ^2^ = 0.11, *p* = 0.02) and SMRA across all levels (T10: *r* = 0.64, *R* ^2^ = 0.41, *p* = 0.0076; T11: *r* = 0.85, *R* ^2^ = 0.72, *p* = 0.0003; T12: *r* = 0.62, *R* ^2^ = 0.38, *p* = 0.0327). SMA at T11 had a negative correlation with VE/VCO_2_ (*r* = −0.36, *R* ^2^ = 0.13, *p* = 0.01). There was no correlation between the number of sternotomies and exercise parameters. Subjects with a pacemaker demonstrated significantly lower peak VO_2_ (*p* = 0.04) and VO_2_ at the anabolic threshold (*p* = 0.03) compared with ACHD patients without a pacemaker.

**Conclusions:**

Abnormal skeletal muscle parameters observed on thoracic cross‐sectional imaging are associated with diminished exercise capacity in ACHD patients. Assessment of thoracic skeletal muscle characteristics may enable early detection of muscle loss, providing valuable insights into the complex factors contributing to exercise limitations in this population.

## 1 Introduction

The population of adults with congenital heart disease (ACHD) continues to grow, accompanied by the natural effects of aging [1]. Medical and surgical advancements have significantly improved life expectancy and quality of life for ACHD patients. However, despite these gains, most ACHD patients experience lifelong morbidities, including reduced exercise capacity [2, 3].

Sarcopenia is a condition marked by a progressive generalized loss of skeletal muscle mass and strength, typically associated with aging or disease. It is linked to an increased risk of physical disability, poor quality of life and mortality [4, 5]. Sarcopenia is an important predictor for heart failure and hospitalization in ACHD patients with Fontan circulation [3]. In addition, sarcopenia has been evaluated in both normal aging individuals and adults with various medical conditions [5, 6, 7]. This study examines sex‐specific thoracic skeletal muscle characteristics in ACHD patients using thoracic advanced imaging techniques and comparing them with established reference values from a healthy historical cohort [8]. Additionally, we investigate their association with exercise capacity.

## 2 Methods

### 2.1 Study Population

This single‐center, retrospective cohort study was approved by the Institutional Review Board (IRB). We reviewed the electronic medical records of ACHD patients aged 18–50 years who underwent cardiopulmonary exercise testing (CPET) and computed tomography (CT) and/or magnetic resonance imaging (MRI) of the chest/heart between 01/01/2010 and 07/31/2021 at the University of Iowa Hospitals and Clinics. The study protocol received IRB approval with a waiver of documentation of informed consent. Patients were excluded if (i) their thoracic CT/MRI studies did not adequately scan the thoracic spine (T10–T12 vertebra) region, (ii) time between their thoracic CT/MRI scan and CPET exceeded 12 months or (iii) they had one or more of the following documented medical conditions which could affect skeletal muscle mass: cerebral palsy, malignancies, autoimmune diseases, diabetes mellitus, autistic spectrum disorders, thoracolumbar spinal trauma affecting paraspinal musculature and chronic inflammatory disorders.

### 2.2 Skeletal Muscle Mass Measurement Using Thoracic CT/MRI

Our institution performs routine CT and/or MRI scans for detailed assessment of the heart and thoracic great vessels in ACHD patients as part of their clinical care. MRI‐derived skeletal muscle area (SMA) strongly correlates with CT measurements at the abdominal level, indicating interchangeability between these modalities [9]. Additionally, body composition measurements from CT and MRI demonstrate strong correlations with dual‐energy X‐ray absorptiometry (DEXA), bioelectrical impedance analysis and cadaveric evaluation, validating their accuracy [10, 11, 12].

Thoracic CTs/MRIs included in the study were non–contrast‐enhanced and obtained within 12 months of CPET. Cardiothoracic CT scans were performed using Somatom Force, Definition or Drive (Siemens, Erlangen, Germany). Cardiac MRIs were performed using either a 1.5 T Magnetom Avanto or Aera (Siemens, Erlangen, Germany). Thoracic CT/MR images were retrieved from the CARESTREAM PACS system. SMA was measured using Vitrea Core software (Canon Medical, Minnetonka, MN, USA) by manually tracing an ellipse region of interest (ROI), which displays mean Hounsfield units (HU; in CT) or pixels (in MR), standard deviation and area after tracing was complete. Skeletal muscle index (SMI)—a heuristic that normalizes muscle area for height—was computed as SMA divided by height‐squared [13]. We used HU values to estimate muscle fat content as they measure tissue attenuation relative to water and allow determination of tissue type and density. Slices in the axial plane nearest to the inferior aspect of thoracic T10, T11 and T12 vertebral bodies were used with the area of pixels within −29 to +150 HU, which is the range of HU containing intramuscular adipose tissue, as previously validated [8, 14, 15]. Skeletal muscle radiation attenuation (SMRA), a measure of muscle fat content in CT and associated with physical function [14, 16, 17, 18], was computed as the mean HU value of all pixels included in SMA [15, 19, 20]. These values were compared with healthy population reference values of similar age (used as a control) reported for sarcopenia assessment including sex‐specific SMA, SMI, SMRA and sarcopenia cutoff values at the same vertebral levels [8].

**FIGURE 1:**
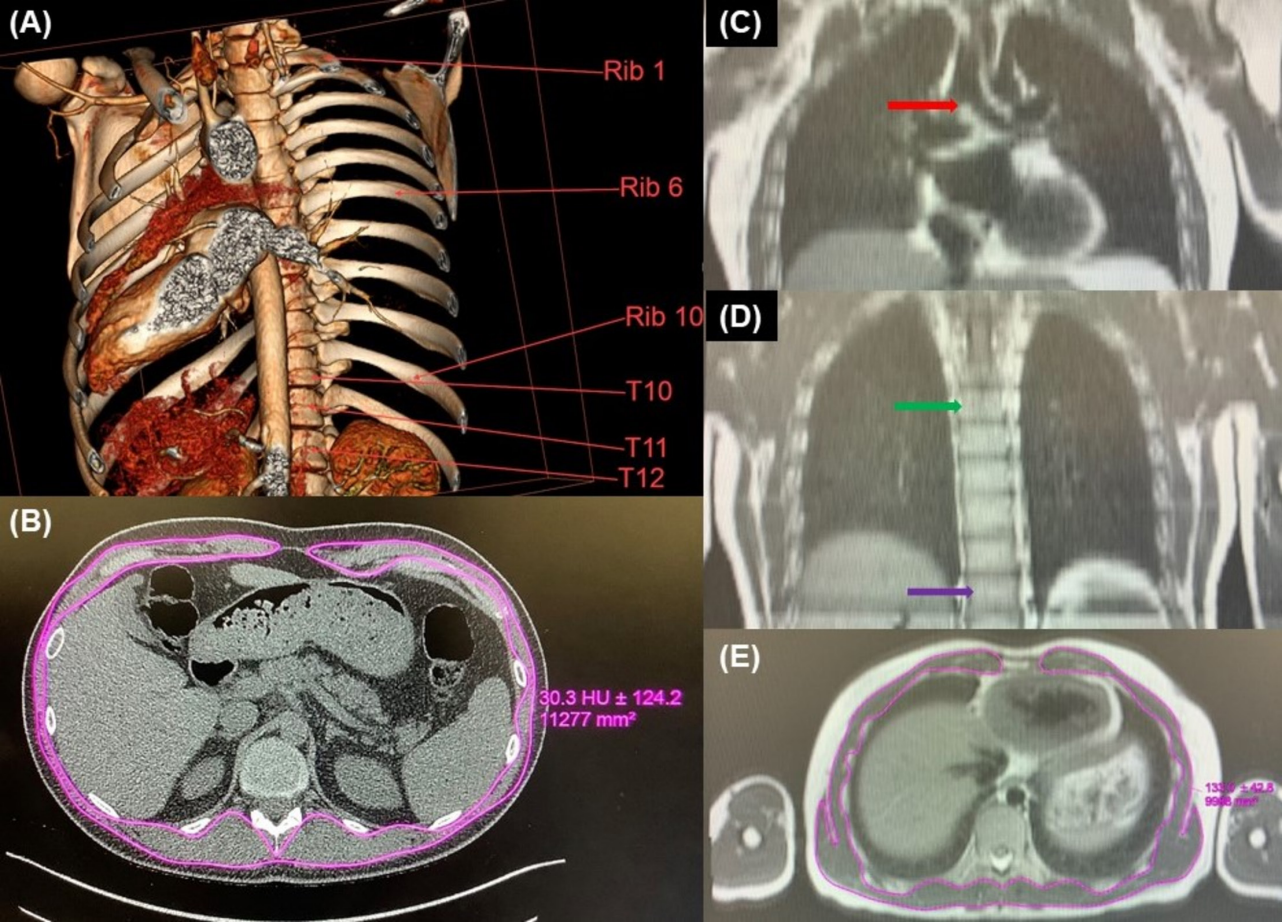
CT/MRI vertebrae numbering and SMA measurement method: In patients with thoracic CT, (A) the ribs and vertebrae were counted from the 3D model and (B) skeletal muscle area (SMA) was calculated by manually drawing using the ROI at the corresponding axial slice at the inferior border of the vertebrae. In an MRI, (C) the carina, shown by the red arrow, can be found, (D) anterior to T5, shown by the green arrow. (D) The vertebrae can then be calculated from T5, e.g., T10 is shown by the purple arrow. (E) shows the traced SMA from MRI.

If CT or MRI scans did not reach the lower T11 and/or T12 levels, only the T10 level slice was included in the study. This was the case for many MRI scans, where the lowest level of the scan only included T10. For CT scans, vertebrae levels were identified by tracing from the posterior end of thoracic rib 1 (connected to T1) and counting down to rib 10 (connected to T10). In MRI studies, vertebrae levels were determined using an alternative approach because of less distinct bony landmarks. The tracheal carina, where the trachea splits into two, as shown in Figure 1, was used as a landmark as it is anterior to T5, and vertebrae were then counted to T10. This method is slightly less accurate than the CT method, since the carina location can differ with breathing.

### 2.3 Cardiopulmonary Exercise Testing (CPET)

CPET in ACHD patients was conducted using the ParvoMedics TrueOne 2400 breath‐by‐breath analysis metabolic measurement system (Salt Lake City, UT) and the Quinton Q‐Stress Cardiac Science treadmill system. Patients performed a maximal‐effort exercise test following the standard Bruce protocol, which involves progressively increasing treadmill speed and incline.

Exercise parameters analysed included peak oxygen consumption (peak VO_2_), peak O_2_ pulse (peak VO_2_ divided by peak heart rate), percent predicted VO_2_ and ventilatory efficiency (VE/VCO_2_ slope), calculated as the ratio of ventilation per unit of carbon dioxide (CO_2_) production.

### 2.4 Statistical Methods

Statistical analyses were conducted using GraphPad Prism version 9.1.2 for Windows (GraphPad Software, San Diego, CA, USA, www.graphpad.com). Continuous variables are presented as mean ± standard deviation, whereas categorical variables are reported as counts (percentage), as appropriate. Continuous variables were analysed using Student's *t*‐test. For all analyses, a *p*‐value of < 0.05 was considered statistically significant. Pearson correlation coefficients (*r*) were calculated to assess relationships between variables.

Skeletal muscle parameters were examined for correlations with exercise parameters and the number of prior sternotomies. We also analysed subgroup populations based on diagnosis, as well as whether the subject had a pacemaker or not.

Intraobserver and interobserver variability in SMA measurement was assessed using Pearson correlation coefficients between two independent readers (J.D.S. and R.A.) at two separate time points, spaced more than 60 days apart.

## 3 Results

### 3.1 Cohort

A total of 60 patients met the inclusion criteria, with cohort details outlined in Table 1. Tetralogy of Fallot was the most prevalent diagnosis, affecting 25% of the cohort. The mean age was 28.3 ± 8.3 years with 52% being males. A total of 18 CT and 42 MRI scans were analysed. Seven patients in the cohort had a pacemaker. Among the 60 patients, 54 (90%) had undergone sternotomies, with a total of 124 procedures performed across the cohort.

**TABLE 1:** Study cohort details.

Total subjects	60
Sex	Males: 31 (52%) Females: 29 (48%)
Age (years) (mean ± SD)	28.3 ± 8.3
CT scans	18
MRI scans	42
Pacemaker	7
Number of patients with sternotomies	54
Diagnosis	
Tetralogy of Fallot	15
Pulmonary atresia/stenosis	8
Dextro‐transposition of the great arteries (TGA) status post arterial switch operation	7
Ebstein's anomaly	7
TGA status post atrial switch operation	5
Aortic stenosis/insufficiency	3
Coarctation of aorta	3
Fontan	3
Atrioventricular (AV) canal defect	2
Levo‐TGA	2
AV valvular disease	1
Double outlet right ventricle	1
Partial anomalous pulmonary venous return	1
Shone's complex	1
Truncus arteriosus	1

The reference population comprised 735 healthy kidney donors who had undergone CT imaging, with 56% identified as male [8]. The mean age was 31.2 ± 6.1 years for females and 30.9 ± 6.1 years for males. For comparative analysis, subjects were sex‐matched to the corresponding individuals within the healthy donor cohort.

### 3.2 Intraobserver and Interobserver Variability

**FIGURE 2:**
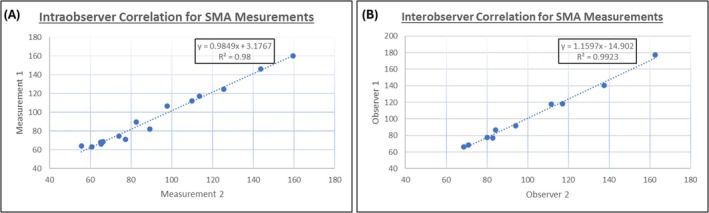
Intraobserver correlation and interobserver variability in skeletal muscle area (SMA) measurement. (A) Intraobserver correlation between two measurements by observer J.D.S. (B) Interobserver correlation between two observers (authors J.D.S. and R.A).

Measurement of SMA demonstrated excellent reliability, with strong intraobserver correlation (*r* = 0.99, *R* ^2^ = 0.98, *p* < 0.0001) and interobserver correlation (*r* = 0.9961, *R* ^2^ = 0.9923, *p* < 0.0001) using the methods described in our study (Figure 2).

### 3.3 Exercise Testing

The mean peak VO_2_ was 26.75 ± 7.3 mL/kg/min, whereas the average peak O_2_ pulse measured 11.7 ± 3.5 mL/beat. The mean VE/VCO_2_ slope was 32.4 ± 4.6, and the average percent predicted VO_2_ was 71.6% ± 17.0%. No significant differences in exercise testing were observed among ACHD subgroups based on diagnosis.

### 3.4 Skeletal Muscle Characteristics

Sex‐specific skeletal muscle parameters are summarized in Table 2. At the T10 and T11 vertebral levels, males exhibited significantly lower SMA (T10: 116.8 ± 24.6 cm^2^, *p* < 0.0001; T11: 114.4 ± 24.7 cm^2^, *p* = 0.0002) and SMI (T10: 37.2 ± 8 cm^2^/m^2^, *p* = 0.005; T11: 36.4 ± 8.1 cm^2^/m^2^, *p* = 0.0014) compared with the healthy reference population. Females had significantly lower SMA at T10 (79.7 ± 14.9 cm^2^, *p* = 0.0242) and T12 (74.2 ± 10.7 cm^2^, *p* = 0.0015) levels, with a trend towards significantly lower SMA at T11 (77.2 ± 11.4 cm^2^, *p* = 0.0565), although statistical significance was not reached. However, female SMI did not differ significantly from the healthy population. Additionally, females displayed significantly lower SMRA at T10 (16.3 ± 14.6 HU, *p* = 0.001), T11 (17.1 ± 10.3 HU, *p* < 0.001) and T12 (25 ± 10.7 HU, *p* < 0.001) compared with both the healthy reference population and males with ACHD. No significant differences in skeletal muscle parameters were found between ACHD subgroups based on diagnosis.

**TABLE 2:** Thoracic skeletal muscle characteristics.

	Thoracic skeletal muscle parameter	ACHD		Control		*p*
		Average	Standard deviation	Average	Standard deviation	
Males	T10 SMA (cm^2^)	116.8	24.6	135.3	22.0	**< 0.0001**
	T10 SMI (cm^2^/m^2^)	37.2	8.0	42.2	6.7	**0.0050**
	T10 SMRA (HU)	41.0	25.2	43.8	5.7	0.7516
	T11 SMA (cm^2^)	114.4	24.7	131.7	21.8	**0.0002**
	T11 SMI (cm^2^/m^2^)	36.4	8.1	41.1	6.8	**0.0014**
	T11 SMRA (HU)	45.4	20.2	46.5	5.3	0.8778
	T12 SMA (cm^2^)	124.7	20.9	141.2	24.4	0.0828
	T12 SMI (cm^2^/m^2^)	39.7	8.3	44.1	7.7	0.2174
	T12 SMRA (HU)	44.3	17.9	48.2	5.3	0.5878
Females	T10 SMA (cm^2^)	79.7	14.9	86.6	16.0	**0.0242**
	T10 SMI (cm^2^/m^2^)	31.0	5.6	32.3	5.9	0.3998
	T10 SMRA (HU)	16.3	14.6	40.4	6.9	**< 0.000001**
	T11 SMA (cm^2^)	77.2	11.4	83.5	15.5	0.0565
	T11 SMI (cm^2^/m^2^)	30.0	3.9	31.0	5.9	0.4240
	T11 SMRA (HU)	17.1	10.3	43.4	6.5	**< 0.000001**
	T12 SMA (cm^2^)	74.2	10.7	91.3	17.6	**0.0015**
	T12 SMI (cm^2^/m^2^)	30.6	4.1	34.0	6.6	0.0955
	T12 SMRA (HU)	25.0	10.7	44.0	6.4	**< 0.000001**

*Note:* Thoracic skeletal muscle characteristics comparison to healthy reference population data. The skeletal muscle characteristics shown are skeletal muscle area (SMA), skeletal muscle indexed to height squared (SMI) and skeletal muscle radiation attenuation (SMRA). SMRA is measured in Hounsfield units and is inversely proportional to fat content in muscle.

### 3.5 Exercise Parameters and Skeletal Muscle Characteristics

**FIGURE 3:**
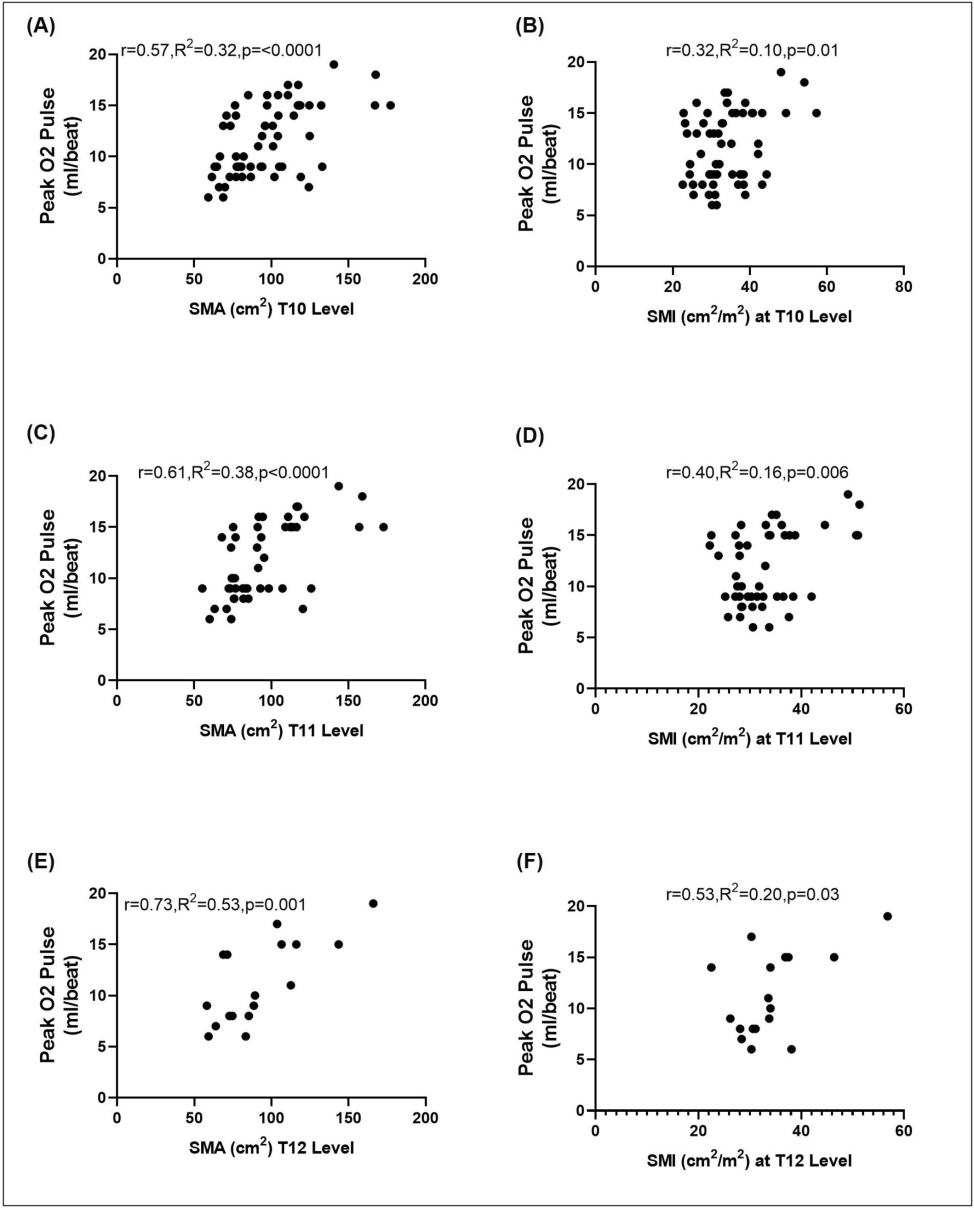
Skeletal muscle characteristics and its correlation with exercise parameters: Correlation between peak O_2_ pulse and skeletal muscle area (SMA; A,C,E) and skeletal muscle index (SMI; B,D,F) at various thoracic vertebral levels in adults with congenital heart disease.

**FIGURE 4:**
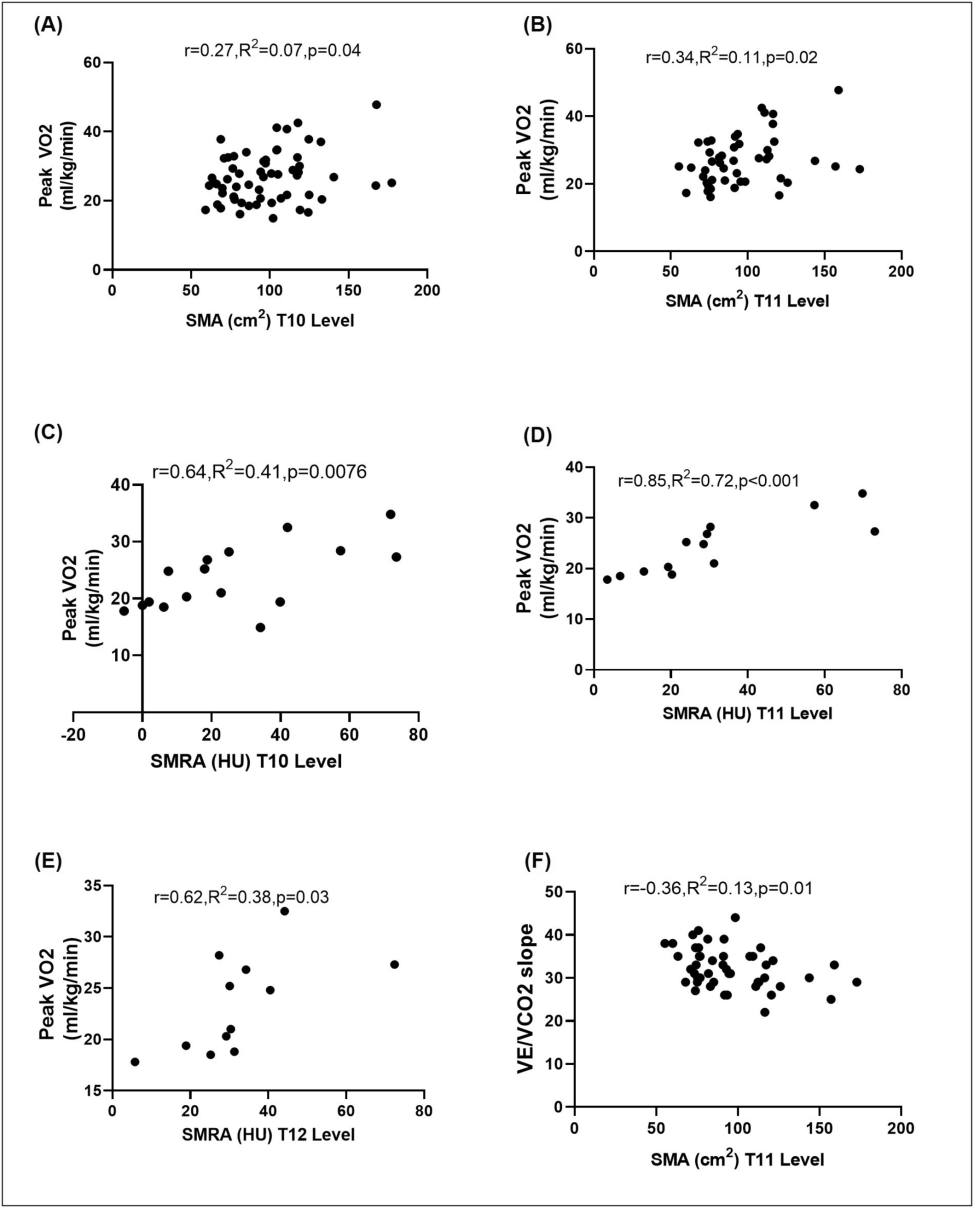
Skeletal muscle characteristics and its correlation with exercise parameters: Correlation between peak VO_2_ and skeletal muscle area (SMA; A,B) and skeletal muscle radiation attenuation (SMRA; C,D,E) at various thoracic vertebral levels in adults with congenital heart disease. (F) Correlation between VE/VCO_2_ slope and SMA at T11 level.

Peak O_2_ pulse demonstrated significant correlations with SMA at T10 (*r* = 0.57, *R* ^2^ = 0.32, *p* ≤ 0.0001; Figure 3A), T11 (*r* = 0.61, *R* ^2^ = 0.38, *p* < 0.0001; Figure 3C) and T12 (*r* = 0.73, *R* ^2^ = 0.53, *p* = 0.001; Figure 3E). Similar correlations were observed between peak O_2_ pulse and SMI (Figure 3B,D,F). Peak VO_2_ correlated with SMA at T10 (*r* = 0.27, *R* ^2^ = 0.07, *p* = 0.0394; Figure 4A) and T11 (*r* = 0.34, *R* ^2^ = 0.11, *p* = 0.02; Figure 4B), as well as with SMRA at T10 (*r* = 0.64, *R* ^2^ = 0.41, *p* = 0.0076; Figure 4C), T11 (*r* = 0.85, *R* ^2^ = 0.72, *p* = 0.0003; Figure 4D) and T12 (*r* = 0.62, *R* ^2^ = 0.38, *p* = 0.0327; Figure 4E). VE/VCO_2_ exhibited a weak but significant correlation with SMA at T11 (*r* = −0.36, *R* ^2^ = 0.13, *p* = 0.01; Figure 4F). No significant correlations were observed between percent predicted VO_2_ and skeletal muscle characteristics.

There was no correlation between the number of sternotomies and exercise parameters (Peak VO_2_: *r* = −0.021, *R* ^2^ = 0.0004, *p* = 0.9779; Peak O_2_ Pulse: *r* = 0.0406, *R* ^2^ = 0.0016, *p* = 0.7625; VE/VCO_2_ slope: *r* = 0.0571, *R* ^2^ = 0.0038, *p* = 0.6437). However, subjects with a pacemaker demonstrated significantly lower peak VO_2_ (*p* = 0.04) and VO_2_ at anabolic threshold (*p* = 0.03) compared with ACHD patients without a pacemaker.

## 4 Discussion

This study highlights the potential utility of cross‐sectional imaging‐based parameters in predicting exercise capacity among ACHD patients.

Our study suggests that ACHD patients have significantly different skeletal muscle characteristics when compared with healthy individuals. When compared with the healthy population, both males and females with CHD experienced lower SMA. However, when normalized for height, females seem to be less affected by their heart condition. Skeletal muscle fat is known to increase with age and have effects on the mechanics of skeletal muscles [21, 22]. Females exhibited significantly lower SMRA at T10, T11 and T12 compared with both healthy controls and male ACHD patients, suggesting greater skeletal muscle fat accumulation in females with ACHD. Female healthy controls are known to have a higher proportion of body fat when compared with healthy males [23]. This may potentially explain why females seem to be less affected in SMI. Their muscle area is not decreased, but their muscle composition has more fat content compared with healthy controls. This is a particularly interesting finding in that the same disease is affecting males and females very differently. These sex‐specific differences may be influenced by hormonal factors, as healthy females naturally exhibit higher body fat percentages than males. Congenital heart disease may further amplify this disparity, contributing to increased muscle fat accumulation in female ACHD patients. Cardiac output and a growth hormone called insulin‐like growth factor‐1 were found to be positively correlated in patients palliated with the Fontan procedure [24]. Also, multiple neurohormonal levels have been found to be elevated in ACHD [25], contributing to the already significant difference between male and female hormones. We used non–contrast‐enhanced CT scan sequences, as previous studies have shown that intravenous contrast significantly affects SMRA values but not SMA [8, 15, 26]. SMA, SMI and SMRA all seem to predict a decreased exercise capacity. Utilizing CT or MRI scans to assess skeletal muscle parameters provides an invaluable alternative for evaluating exercise capacity risks in ACHD patients. This approach could aid clinicians and patients in developing targeted interventions to mitigate functional decline. The results also indicate that skeletal muscle mass and exercise capacity should be assessed in these patients to help risk‐stratify, as previous studies have shown that poor exercise capacity predicts hospitalization or death [27]. This is especially important in patients who may not be able to undergo CPET. Clinically, routine skeletal muscle imaging can inform risk stratification, guide exercise and rehabilitation recommendations and monitor the progression of frailty. This is especially important for tailoring interventions and predicting outcomes in patients with complex CHD [28]. Advanced modalities such as magnetic resonance spectroscopy (MRS) can further assess muscle bioenergetics, providing insight into metabolic adaptations in hypoxemic states and heart failure [29].

Subgroup analysis by type of CHD was done on CHD populations with at least five subjects, which included patients with tetralogy of Fallot, pulmonary atresia/stenosis, Ebstein's anomaly, atrial switch or arterial switch. None of the five CHD subgroups exhibited statistically significant differences in the skeletal muscle or exercise parameters compared with the overall cohort, likely due to the limited sample size. Further studies with larger populations are necessary to validate these findings. Seven of our subjects had pacemakers. Lowered exercise parameters in these subjects could be due to pacemaker parameters or associated low ventricular ejection fraction.

Exercise capacity, as assessed by peak VO_2_, has been found to be reduced in ACHD patients [27, 30] and strongly correlated with the risk of hospitalization or death [27]. The peak VO_2_ estimates the peak combined aerobic performance of the cardiac, pulmonary and muscular systems. In our study, peak VO_2_ correlated significantly with thoracic skeletal muscle characteristics, specifically area and fat content. Low skeletal muscle mass correlates with poorer peak VO_2_ in adult patients [2, 31]. Our study provides similar findings, suggesting that an increase in thoracic SMA and a decrease in its fat content might correlate with improved peak VO_2_ in ACHD patients. Peak O_2_ pulse, a marker of stroke volume and oxygen extraction, correlated with SMA and SMI at T10, T11 and T12 levels. The amount of abdominal (L3 vertebral level) skeletal [32] and appendicular lean mass positively correlated with oxygen pulse in adult Fontan patients, probably reflecting a superior peripheral pump and healthy muscles [2]. In this study, we derive similar results in all ACHD groups including those patients with Fontan physiology, using thoracic skeletal muscle characteristics. Ventilatory efficiency (VE/VCO_2_ slope) is raised in all ACHD groups and is another commonly used predictor of morbidity and mortality [33]. The VE/VCO_2_ slope was expectedly higher (32.4 ± 4.6) in our patients when compared with the reported normal controls (25.6 ± 3.1) in the study by Dimopoulos K. et al. [33]. Skeletal muscle mass is an independent predictor of VE/VCO_2_ slope [31]. Most of the previous studies investigating exercise capacity and skeletal muscle mass have used DEXA scan to measure skeletal muscle mass [2, 31]. We provide similar data but in ACHD patients by analysing thoracic skeletal muscle using previously acquired clinical CT/MRI of the chest or heart.

Respiratory muscle weakness also leads to restricted ventilation in ACHD patients, which leads to reduced exercise capacity [34]. Diaphragm weakness may compound skeletal muscle loss, further impairing exercise capacity. Additionally, weakened respiratory function could contribute to muscle deterioration, exacerbating exercise intolerance in ACHD patients. The correlation between skeletal muscle loss and diaphragm weakness together should be studied in the future.

ACHD patients with thoracic skeletal muscle wasting could benefit from targeted interventions, including respiratory muscle training and specialized nutritional therapy aimed at preserving muscle mass. Further studies are required to explore these strategies as potential treatments for exercise limitations in this population. Moreover, exercise limitation is just one of the multiple comorbidities ACHD patients experience. Our study shows how the implications of congenital heart disease are much broader than the heart, and the complex physiology of this population and its effects on all body systems should be studied.

Routine skeletal muscle imaging enables early detection of muscle pathology, monitoring of disease progression and assessment of treatment response [35, 36, 37]. For sarcopenia, imaging modalities (DXA, CT, MRI and US) are used to assess muscle mass and quality and are predictive of adverse outcomes such as falls, disability and mortality [38, 39]. Future directions include establishing validated imaging thresholds for muscle quality, expanding routine use in clinical practice and integrating point‐of‐care ultrasound. Ongoing research is needed to standardize protocols and improve accessibility, with the goal of making skeletal muscle imaging a routine part of patient management across diverse clinical settings including ACHD care [28, 37, 40].

### 4.1 Limitations

This study has inherent limitations due to its single‐centre, retrospective and cross‐sectional study design. Cohort size was constrained, particularly in the assessment of SMRA from CT scans. Additionally, the specific type of congenital heart disease likely influences the skeletal muscle measurements, but limited subgroup sample sizes precluded detailed analysis. MRI‐derived skeletal muscle measurements were compared with reference population CT scans [8]; however, agreement between these modalities has only been validated at a specific anatomical level [9].

## 5 Conclusions

ACHD are at increased risk for skeletal muscle loss, which may contribute to diminished exercise capacity. Integrating thoracic skeletal muscle assessment into routine advanced non‐invasive imaging of the heart and chest could facilitate early detection of skeletal muscle deterioration. This approach may improve our understanding of the multifactorial contributors to exercise limitations in ACHD patients, ultimately informing strategies for intervention and management.

## Funding

The authors have nothing to report.

## Ethics Statement

The study was approved by the Institutional Review Board (IRB) of the University of Iowa (IRB ID # 202003159). The study protocol received IRB approval with a waiver of documentation of informed consent.

## Conflicts of Interest

The authors declare no conflicts of interest.
